# Reference Gene Selection for Quantitative Real-Time PCR Normalization in Larvae of Three Species of Grapholitini (Lepidoptera: Tortricidae)

**DOI:** 10.1371/journal.pone.0129026

**Published:** 2015-06-01

**Authors:** Jaryd A. Ridgeway, Alicia E. Timm

**Affiliations:** Department of Zoology and Entomology, Rhodes University, Grahamstown, South Africa; Institute for Sustainable Plant Protection, C.N.R., ITALY

## Abstract

Despite the agricultural importance of species in the Grapholitini (Lepidoptera: Tortricidae), and the value of gene expression analysis for improved population management, few gene expression studies based on quantitative real-time PCR (qPCR) have been conducted for this tribe. Part of the reason for this lack of information is that suitable reference genes, which are fundamental for accurate normalization of qPCR studies, have not been identified for the tribe. Thus, the expression stability of six potential reference genes (ACT, AK, COI, EF1, ENO and TUB) was assessed in three different tissues (whole body, midgut and cuticle) of *Cryptophlebia peltastica* (Meyrick), *Cydia pomonella* (L.) and *Thaumatotibia leucotreta* (Meyrick). Additionally, these reference genes were tested using *T*. *leucotreta* at different temperatures (15°C, 25°C and 35°C) with and without baculovirus infection. Suitable reference genes were identified for the whole body and midgut tissue of all three species, and for cuticle tissue of *Cy*. *pomonella* and *T*. *leucotreta*. When *T*. *leucotreta* was infected with the virus at all temperature conditions ACT, AK and EF1 were found to be the most suitable reference genes for experimental normalization. In general, for all tissue types, species and stress conditions, AK and EF1 were the best-performing reference genes. However, even though the three species analysed were closely related and within the same tribe, each species required varying gene combinations for suitable normalization. This study provides the first reference gene evaluation for the Tortricidae, and paves the way for future qPCR analysis in Tortricidae.

## Introduction

Insect gene expression studies have increased considerably during the last five years. The major influencing factor for this trend is the availability of genomic information paralleled with high throughput, robust and accurate gene expression analysis tools such as real-time quantitative PCR (qPCR) [1, 2]. In the agricultural sector these gene expression studies can provide crucial data to improve production. One such example is using gene expression to identify and quantify key genes involved with virus interactions, that can then be used to predict the success of biological control agents or improve current control measures [2]. As the use of biopesticides is increasing, genetic clues for improving the efficacy of these control measures will not only benefit production but also provide a more competitive and reliable alternative to chemical controls.

Although qPCR is the current gold standard for gene expression analysis, it has been recognised as having high variability if the recommended steps are not taken for proper normalization [3, 4]. As with all basic experimental processes, results of the identified treatments are dependent on the stability of the controls. The importance of qPCR normalization has been reviewed several times [2, 4, 5]. However, as insect gene expression studies using qPCR are still relatively recent, the importance of accurate normalization cannot be overstated. No universal reference genes for insects have been identified yet as there are many different factors that influence gene expression and the subsequent stability of reference genes [2, 6, 7]. It is therefore crucial to assess reference genes for the same experimental conditions under which the target genes will be evaluated. Factors known to influence gene stability at the species level are temperature [8], virus infection [1], pesticide exposure [2], tissue type [9], geographic group [5], age [10], sex [11] and diet [12]. For normalization of qPCR, reference genes should provide consistent expression across all conditions. The qPCR process would be simplified if the same genes and primers could be used for different species. However, information about taxonomic trends for reference gene selection is limited. In a study examining reference genes in Lepidoptera, no genes were found that could be used as universal reference genes [7]. Reference gene evaluation at lower taxonomic levels may still reveal evidence for trends in closely related species.

This study analysed species within the tribe Grapholitini (Lepidoptera: Tortricidae), which contains some of the world’s most destructive horticultural pests. Three pest species of major economic importance were included in the analysis—*Cryptophlebia peltastica* (Meyrick), *Thaumatotibia leucotreta* (Meyrick) and *Cydia pomonella* (L.). Currently both *T*. *leucotreta* and *Cy*. *pomonella* are controlled using commercially-produced granulovirus formulations and research is underway for the formulation of a virus of *C*. *peltastica*. In the last decade *Cy*. *pomonella* has developed resistance to the granulovirus used in its control [13–15]. This led to concerns that *T*. *leucotreta* and *C*. *peltastica* may also develop resistance to granuloviruses, due to the close relatedness of the three insect species and of their granuloviruses [16, 17]. Thus, establishing a foundation for future gene expression studies using Grapholitini is necessary.

The aim of this study was to evaluate potential reference genes for species within the Grapholitini. Reference gene evaluation was performed for *C*. *peltastica*, *T*. *leucotreta* and *Cy*. *pomonella* to provide a comparison for reference gene selection among species that are closely related, which might provide an indication of possible trends at fine taxonomic levels. For each species, the whole bodies, midgut and cuticle tissue were compared to examine their effect on reference gene selection [18, 19]. The three tissue types chosen for reference gene evaluation have advantages for a variety of future gene expression studies. In addition, the influence of biological stress on reference genes was examined by exposing *T*. *leucotreta* to the granulovirus used for its control at different temperatures. Temperature has been observed to have a major influence on the efficacy of viruses to control their hosts’ populations [20, 21]. Results provided by this study will be fundemental for further qPCR studies on gene expression in *T*. *leucotreta*, *C*. *peltastica* and *Cy*. *pomonella* and the potential improvement for their control.

## Material and Methods

### Insect material

The three species used in this study—*C*. *peltastica*, *T*. *leucotreta and Cy*. *pomonella*—were obtained from established cultures at River Bioscience (Pty) Ltd. The larvae were received as 1^st^ instars with their species-specific artificial diet formulation. The *C*. *peltastica* and *T*. *leucotreta* diets consisted of 40 g maize meal, 4 g wheat germ, 0.73 g milk powder, 0.13 g sorbic acid, 0.3 g methyl paraben, 2g brewer’s yeast and 45 ml type 1 distilled water. The *Cy*. *pomonella* diet consisted of 0.36 g nipagin, 0.46 g benzoic acid, 28.24 g whole wheat flower, 7.11 g wheat germ, 7.57 g yeast, 1 g ascorbic acid, 0.28 ml of formalin and 45 ml type 1 distilled water. Individual larvae were placed in separate Petri dishes with a 5mm x 20mm x 20mm diet cube and reared at a constant temperature of 25°C. The diet was rehydrated, using type 1 distilled water, every 48 h. As stable gene expression is highly dependent on the age of the animal [22, 23], only 4^th^-instar larvae, selected based on head capsule size in accordance with Dyar’s rule [24], were used in analyses to ensure that results could be meaningfully compared. The 4^th^-instar larvae were examined as they were large enough to dissect and measure virus uptake accurately while still being immature enough to not pupate prematurely under stress conditions.

### Tissue comparison

Three different tissue compositions—midgut, cuticle and whole body—were used for each of the three species to test the effect of tissue composition on reference gene stability. Dissections of the midgut and cuticle tissue were performed in insect Ringer solution [25] and dissected tissue was immediately placed in either Qiagen RNAlater RNA stabilization reagent (Qiagen, Germantown, USA) or flash-frozen in liquid nitrogen, then stored in a -80°C freezer until RNA extraction. Three biological replicates consisting of a pool of eight individuals per replicate were used for each tissue type for each species.

### Biological treatments

As *T*. *leucotreta* will be used in downstream qPCR experiments, it was the species selected for the biological treatments. All larvae were 4^th^-instar after the termination of each experiment. The required instar for the start of each treatment was determined using preliminary study data.

The *Cryptophlebia leucotreta* granulovirus (CrleGV-SA) [26] was purified as described elsewhere [27, 28] and diluted to a concentration of 10^7^ OBs/ml. This concentration was required to ensure a lethal dosage [29]. Droplet feeding treatments were done using previously described methods [30]. Upon reaching the desired instar, the diet cube was removed from the individual Petri dishes for 12 hours. Thereafter, a droplet of either distilled H_2_O and food colouring mix (for controls) or diluted CrleGV-SA and food colouring (for treatments) was presented to the larvae. The dark blue food colouring provided a visual confirmation of consumption. Larvae were manipulated in a dimly-lit room with a number 2 paint brush to minimize stress. Using a Mettler Toledo XPE 205 Analytical Balance, larvae were weighed before and after consuming the droplet. For each experiment, eight infected and eight control 4^th^-instar *T*. *leucotreta* larvae were selected. After droplet feeding the diet cube was placed back into the Petri dish, which was sealed using a rubber band and placed into an airtight container. Experiments examining the effects of biological stress on reference gene selection in *T*. *leucotreta* were conducted at 15°C, 25°C and 35°C for 48 hours and 25°C for 24 and 72 hours, using CrleGV-infected and uninfected larvae. All experiments were repeated in triplicate, resulting in 15 independent treatments.

### RNA extraction and cDNA conversion

RNA extractions were performed using the SV Total RNA Isolation System (Promega, Madison, USA), as it is known to produce RNA of a good quality and quantity. The SV Total RNA Isolation System incorporates a DNase step during the extraction and therefore further post extraction DNase methods are unnecessary [31]. All tissue was homogenised with liquid nitrogen using a sterile mortar and pestle following the manufacturer’s protocol.

RNA sample quality was tested using the NanoDrop 2000 spectrophotometer (Thermo Scientific, Wilmington, USA). RNA samples were additionally analysed for DNA contamination using agarose gel electrophoresis (AGE), with 1% agarose gels run for 30 mins at 80V. The gels were loaded with a 100bp DNA ladder (Promega, Madison, Wisconsin, USA) as well as two Lambda DNA standards of 20 ng and 100 ng. 5μl of RNA was used per sample. For further confirmation of RNA quality and lack of DNA contamination, a random sample was tested with an Agilent 2100 Bioanalyzer (Agilent Technologies, Palo Alto, USA). The RNA was converted to cDNA using the Maxima H Minus Reverse Transcriptase kit with a random hexamer primer (5’-d(NNNNNN)-3’) (Thermo Scientific, Wilmington, USA). The cDNA concentration was measured using the NanoDrop 2000 spectrophotometer and then diluted to 1000ng/ μl.

### Virus infection confirmation

To verify that control samples were uninfected and that treated samples were infected with CrleGV-SA, a PCR targeting the granulin gene was performed as described elsewhere [32]. The PCR products were electrophoresed on a 1% agarose gel for 30 minutes at 80 V. Random infected samples were further verified for infection by Sanger sequencing conducted by Macrogen using the same primers used in the PCR.

### Selection of candidate reference genes and primer design

Six genes were selected for analysis—Actin 5C (ACT), Elongation factor 1-α (EF1), α-Tubulin (TUB), Arginine kinase (AK), Cytochrome oxidase subunit 1 (CO1) and Enolase (Eno). Primers amplifying the ACT, EF1, TUB genes were obtained from previous studies (Table 1) [33–35]. Primers amplifying the AK, CO1, and ENO (Table 1) genes were designed using conserved regions of multiple Tortricidae sequences (S1 Table). These primers were designed using Primer3 0.4.0 software (Rozen & Skaletsky, 1999) (http://bioinfo.ut.ee/primer3-0.4.0/primer3/). The parameters set for primer design required a melting temperature (Tm) between 60–63°C, GC content of 50–60%, amplified region length 55–190 bp, max 3’ self complementary of 1, max poly-X = 3, primer length between 18 and 20bp, primer paired toward 3’end and a max Tm difference of 10. A ten-times dilution series was performed (1000 ng/μl, 100 ng/μl, 10 ng/μl and 1 ng/μl) using a pool of eight individual *C*. *peltastica*, *T*. *leucotreta* and *Cy*. *pomonella* whole body cDNA with three technical replicates. The CFX Connect (BIO-RAD, Hercules, USA) program provided analysis of amplification efficiencies and melt curve data. For primers to be identified as suitable, they required a melt curve with a single peak to verify the absence of primer-dimers and amplification efficiencies between 90–110% and an R-squared value above 0.95.

**Table 1:** Primers used for reference gene selection for normalization of qPCR analysis of three species of Tortricidae.

				Amplification efficiency % (R^2^)		
Gene	Primer sequence (5'-3')	Amplicon length (bp)	Reference species	*C*. *peltastica*	*Cy*. *pomonella*	*T*. *leucotreta*
ACT	F:AAT TAC CAT TGG TAA CGA GCG ATT, R:TGC TTC CAT ACC CAG GAA TGA	73	*Drosophila melanogaster* [34]	107.25 (0.965)	101.05 (0.987)	104.31 (0.996)
AK	F:CTA AGG AAA CCC AGC AGC AG, R:GGC AGT CAC CAA CCT CTT GT	188	Tortricidae	101.25 (0.985)	106.9 (0.968)	97.85 (0.985)
CO1	F:CCG GGA TCT TTA ATT GGT GA, R:CAT CCT GGT CCT GCT CCA TT	171	Tortricidae	2453.55 (0.955)	104 (0.993)	99.6 (0.994)
EF1	F:ACG TCT ACA AAA TCG GCG GT, R:GAT GGT GGC AGG TGC GAA TA	61	*Epiphyas postvittana* [33]	107.2 (0.999)	105 (0.985)	101.85 (0.995)
ENO	F:ACT TGG TGC TAA CGC CAT TC, R:GCC AAG TCA GCC AAG TGT TT	59	Tortricidae	No amplification	102.5 (0.959)	97.4 (0.987)
TUB	F:ACC CGC GTA TCC ACT TCC C, R:AAC TCG CCC TCC TCC ATA CC	Not available	*Plutella xylostella* [35]	94 (0.939)	96 (0.985)	97.8 (0.986)

All qPCR experiments were conducted using the CFX Connect machine and iTaq Universal SYBR Green Supermix (BIO-RAD, Hercules, USA). The qPCR thermal cycling profile used was set according to a previous study [34]. Species and tissue comparisons used a final reaction volume of 25 μl consisting of 1 μl of each primer (400nM), 10 μl Supermix, 0.25 μl cDNA (250ng) and 12.75 μl H_2_O. The *T*. *leucotreta* stress qPCR reactions comprised of a final volume of 10 μl, consisting of 1 μl cDNA (500 ng), 5 ul Supermix, 0.25 μl of each primer (100nM) and 3.5 μl H_2_O. Three technical replicates were used for each sample and H_2_O replaced template for the non-template controls.

### Analysis software

Raw quantification cycle (Cq) values were captured using the Biogazelle qBase+ software. These were then transferred into additional reference gene analysis programs. The three most widely accepted programs for reference gene evaluation, Bestkeeper [36], geNorm (Biogazelle qBase+) [37] and Normfinder [38], were used to analyse gene expression stability. Bestkeeper provides a geometric mean (CP) and standard deviation (SD) from the samples’ Cq value for each gene. The SE value calculated by Bestkeeper was used to determine if a gene had a SE value below the recommended cut-off value of 1. The geNorm analysis program is based on a similar algorithm to Bestkeeper but it provides a stability value (M) and a coefficient of variation (CV) of the normalized genes selected. The program also suggests the optimal gene pairing of up to six genes with a calculation of pair-wise variation (V). To avoid misleading results due to co-regulation, genes were excluded from the analysis in a step-wise manner to determine the actual values for the selected reference genes. Normfinder identifies stability values and SE of analysed genes. The program also identifies the best gene pair by using inter- and intragroup variation. However, if the sample is only based on one grouping variable, the program can only provide one best performing reference gene. By taking into account the strengths and limitations of all three programs, we provided a selection of suitable reference genes for all of the different analysed conditions.

## Results

### Sample validation

This study conforms with the Minimum Information for Publication of Quantitative Real-Time PCR guidelines [4]. The average RNA integrity number (RIN) of the RNA sub-sampled was 9.87. The mean purity values measured using the NanoDrop 2000 spectrometer were 2.18 ± 0.03 (280/260) and 2.17 ± 0.14 (230/280) (S2 Table). All larvae in this study ingested an average of 1.18 ml (± 0.5ml) of droplet mix. For analysis of *T*. *leucotreta* response to its granulovirus, control treatments tested negative for CrleGV-SA while all treatment samples tested positive. Treated samples that were sequenced showed a 99% match with *C*. *leucotreta* granulovirus isolates (CrleGV-SA).

### Primer optimization and amplification

For all three species, the ACT, AK, EF1 and TUB genes were found to have an expression level below the required value (Cq < 35) with amplification efficiencies between 90–110% and no evidence of primer-dimers; correlation coefficients were all above 0.95. Primers for CO1 and ENO satisfied the conditions above for *Cy*. *pomonella* and *T*. *leucotreta*. However, CO1 for *C*. *peltastica* samples showed poor amplification efficiency and ENO did not amplify. Therefore, the CO1 and ENO genes were excluded from all *C*. *peltastica* analyses.

### Expression levels

**Fig 1:**
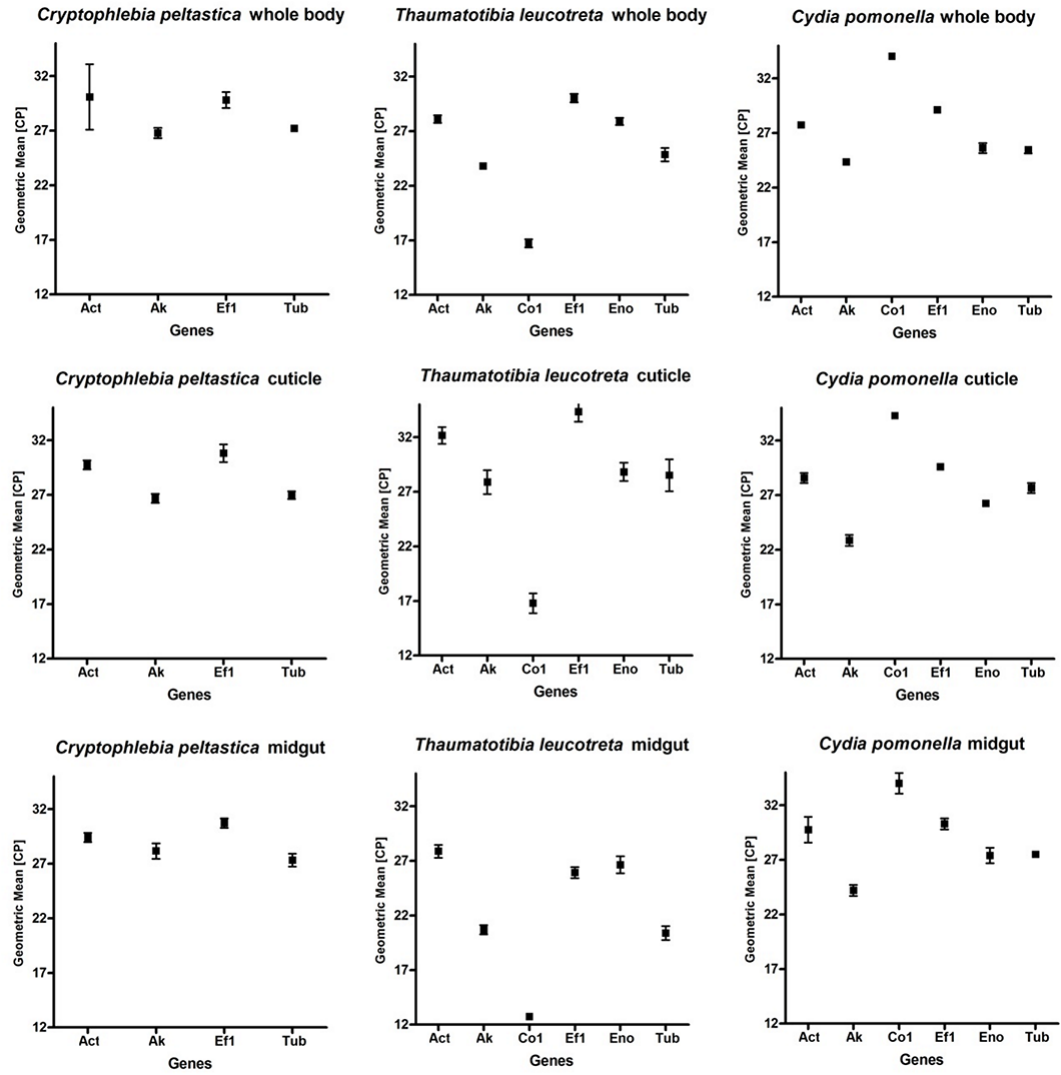
Expression levels for candidate reference genes for three different tissue types and species of Tortricidae. Expression levels of reference genes are expressed in cyclic-threshold (Cp). Boxes indicate Cp means and whiskers standard deviations from the means.

The expression levels of the six genes were all between 12 and 35 CP (Fig 1), calculated as a mean of biological and technical replicates of each sample. High CP values represent low expression levels. The CO1 gene was the most highly expressed gene in all *T*. *leucotreta* samples with a mean CP value of 15.44 (± 2.32) and showed the lowest expression in the *Cy*. *pomonella* samples with a mean expression of 34.1 (± 0.144) (Fig 1). ACT had the highest variability of expression in the *C*. *peltastica* whole body samples with a standard deviation (SD) of ±2.99 (Fig 1). The SDs of the CP values for *T*. *leucotreta* for the different tissue types for the same genes was on average four times higher than the SDs of the other two species. In addition, expression levels for the same genes in the different tissues of *T*. *leucotreta* were more similar than in *C*. *peltastica* and *Cy*. *pomonella*.

### Pair-wise variation

For the majority of species and tissue comparisons, geNorm identified the optimal number of reference genes as two, although *Cy*. *pomonella* midgut samples required four genes for accurate normalization (data not shown). The required number of reference genes varied with *T*. *leucotreta* under stressful conditions. At 15°C and 25°C for both virus-infected and uninfected samples, only two genes were required for normalization. Two genes were also required for normalization of uninfected samples at 35°C. However, when uninfected and infected samples were combined in the analysis at 35°C, four genes were required for normalization. Analysis including all three temperatures, with virus-infected and control samples required three genes for accurate normalization.

### Analysis of gene expression stability

#### Species comparison

For *C*. *peltastica* samples, the Bestkeeper, Normfinder and geNorm programs all identified AK and TUB as the most stable reference genes and the best combination for analysing cDNA generated from whole body RNA extractions (Table 2). ACT was rejected by all three programs as it had high values for SD (±2.99), SE (±1.52) and poor stability (3.02). In the *Cy*. *pomonella* whole body sample, AK and EF1 were the most stable and best gene combination by all three programs (Table 2). Normfinder ranked genes from most stable to least stable as AK, EF1, TUB, CO1, ENO and ACT. In the *T*. *leucotreta* whole body sample Normfinder and geNorm found ACT and COI to be the overall best combination of genes for normalization (Table 2).

**Table 2:** Analysis of candidate reference genes for normalization of *C*. *peltastica*, *Cy*. *pomonella* and *T*. *leucotreta* whole body tissue.

	Bestkeeper analysis	Normfinder analysis		geNorm analysis	
	Standard Deviation (> 1)	Stability value (> 1)	Standard error (> 1)	M (> 0.5)	CV (> 0.3)
***C*. *peltastica* whole body**					
ACT	2.987	3.016	1.519		
AK	0.474	**0.386**	**0.306**	**0.208**	**0.021**
EF1	0.733	0.725	0.235		
TUB	0.182	0.693	0.234	**0.289**	**0.133**
***Cy*. *pomonella* whole body**					
ACT	0.064	0.414	0.222		
AK	0.302	**0.026**	**0.362**	**0.041**	**0.014**
COI	0.159	0.297	0.174		
EF1	0.293	0.026	0.362	**0.041**	**0.014**
ENO	0.459	0.344	0.192		
TUB	0.330	0.221	0.147		
***T*. *leucotreta* whole body**					
ACT	0.358	**0.095**	**0.157**	**0.118**	**0.040**
AK	0.057	0.222	0.123		
COI	0.379	0.210	0.122	**0.118**	**0.041**
EF1	0.391	0.214	0.122		
ENO	0.340	0.726	0.238		
TUB	0.611	0.564	0.193		

Values in bold indicate best performing genes for Normfinder and geNorm analyses. Due to possible misleading results resulting from co-regulation, only the best gene combinations are shown for geNorm analysis.

#### Tissue type comparison

geNorm and Bestkeeper identified only 2 genes that were stable enough to use as reference genes for analyses of midgut tissue for the three species—EF1 and TUB. Although Normfinder also identified EF1 and TUB as the most stable genes, these had a stability above the proposed cut-off value of 1.00. When midgut tissue was analysed separately for each species, all three programs identified suitable reference genes.

For the *C*. *peltastica* midgut samples, geNorm identified AK and TUB as the best gene combination for normalization (Table 3). Normfinder also showed that AK and TUB were suitable reference genes but selected EF1 as the best overall performing gene. All genes were found to be acceptable by Bestkeeper.

**Table 3:** Analysis of candidate reference genes for normalization of *C*. *peltastica*, *Cy*. *pomonella* and *T*. *leucotreta* midgut tissue.

	Bestkeeper analysis	Normfinder analysis		geNorm analysis	
	Standard Deviation (> 1)	Stability value (> 1)	Standard error (> 1)	M (> 0.5)	CV (> 0.3)
***C*. *peltastica midgut***					
**ACT**	0.420	0.416	0.171		
**AK**	0.697	0.590	0.204	**0.108**	**0.037**
**EF1**	0.429	**0.126**	**0.237**		
**TUB**	0.580	0.330	0.162	**0.108**	**0.037**
***Cy*. *pomonella* midgut**					
**ACT**	1.184	1.308	0.477		
**AK**	0.505	**0.114**	**0.503**	**0.439**	**0.040**
**CO1**	0.950	0.910	0.363		
**EF1**	0.514	0.114	0.503	0.662	0.307
**ENO**	0.698	0.629	0.266	0.721	0.318
**TUB**	0.235	0.218	0.316	**0.550**	**0.200**
***T*. *leucotreta* midgut**					
**ACT**	0.590	0.795	0.261		
**AK**	0.420	0.165	0.232	**0.152**	**0.054**
**CO1**	0.290	0.254	0.193		
**EF1**	0.500	0.533	0.208	**0.152**	**0.052**
**ENO**	0.780	1.362	0.402		
**TUB**	0.640	**0.163**	**0.234**		

Values in bold indicate best performing genes for Normfinder and geNorm analyses. Due to possible misleading results resulting from co-regulation, only the best gene combinations are shown for geNorm analysis.

For *Cy*. *pomonella* midgut tissue samples, geNorm required AK, EF1, ENO and TUB to be used in combination for accurate normalization (Table 3). Normfinder found AK to be the best-performing gene with only ACT being rejected due to a stability value of 1.31. Bestkeeper found ACT to have a SD above the cut-off value of 1.

The best gene combination found by geNorm for *T*. *leucotreta* midgut tissue samples was AK and EF1 (Table 3). Although both these genes were found to be acceptable reference genes by Normfinder, TUB was selected as the best-performing gene.

Analysis of the cuticle tissue gene expression values for all three species showed that none of the three programs found genes suitable to be used as reference genes for normalization (data not shown). Therefore, cuticle tissue samples of the individual species were analysed separately.

For *C*. *peltastica* cuticle tissue samples, Bestkeeper found that all genes had acceptable SD values (Table 4). TUB was chosen by Normfinder as the overall best-performing gene. However, it was still not considered to be stable. Thus, Normfinder and geNorm found no genes suitable as reference genes in this sample type.

**Table 4:** Analysis of candidate reference genes for normalization of *C*. *peltastica*, *Cy*. *pomonella* and *T*. *leucotreta* cuticle tissue.

	Bestkeeper analysis	Normfinder analysis		geNorm analysis	
	Standard Deviation (> 1)	Stability value (> 1)	Standard error (> 1)	M (>0.5)	CV (>0.3)
***C*. *peltastica* cuticle**					
ACT	0.422	1.321	1.323		
AK	0.399	1.495	1.345		
EF1	0.822	2.087	1.489		
TUB	0.360	**0.461**	**1.788**		
***Cy*. *pomonella* cuticle**					
ACT	0.422	1.038	0.538	**0.256**	**0.088**
AK	0.501	**0.158**	**0.305**		
CO1	0.249	0.470	0.303		
EF1	0.324	0.483	0.308	**0.256**	**0.088**
ENO	0.071	0.553	0.332		
TUB	0.461	0.566	0.337		
***T*. *leucotreta* cuticle**					
ACT	0.775	0.368	0.215		
AK	1.098	0.099	0.160		
CO1	0.920	**0.026**	**0.399**	**0.015**	**0.005**
EF1	0.908	0.096	0.162	**0.015**	**0.005**
ENO	0.838	0.189	0.157		
TUB	1.462	0.903	0.455		

Values in bold indicate best performing genes for Normfinder and geNorm analyses. No suitable genes were identified for *C*. *peltastica* cuticle by geNorm analysis. Due to possible misleading results resulting from co-regulation, only the best gene combinations are shown for geNorm analysis.

For *Cy*. *pomonella* cuticle tissue samples, geNorm identified ACT and EF1 as the best-suited gene pair for normalization (Table 4). In contrast, Normfinder rejected ACT as it had a stability value of 1.038. Normfinder found AK to be the best-performing gene and ranked the genes from most to least stable as AK, CO1, EF1, ENO, TUB and ACT. Bestkeeper found that all genes satisfied the SD thresholds.

For *T*. *leucotreta* cuticle samples, all three programs selected CO1 and EF1 as the most stable genes with CO1 0.03 higher than ENO making it the third most stable gene combination (Table 4). Bestkeeper rejected TUB and AK as potential genes due to their SD being above 1.

#### Biotic stress effects on Thaumatotibia leucotreta

When all stress conditions (15°C, 25°C and 35°C with virus-infected and uninfected midgut tissue over 24, 48 and 72 hour periods) for *T*. *leucotreta* were analysed in combination geNorm selected ACT, AK and EF1 as suitable genes for normalization (Table 5). Normfinder identified AK and EF1 as the best gene pair with ACT as the third best-performing gene.

**Table 5:** Analysis of candidate reference genes for qPCR normalization of *T*. *leucotreta* under different biological conditions, including CrleGV infection at three different temperatures.

	Bestkeeper analysis	Normfinder analysis		geNorm analysis	
	Standard Deviation (> 1)	Stability value (> 1)	Standard error (> 1)	M (>0.5)	CV (>0.3)
**15**°**C uninfected**					
ACT	0.280	0.133	0.371		
AK	0.150	**0.087**	**0.507**	**0.047**	**0.016**
CO1	0.280	0.373	0.294	**0.047**	**0.016**
EF1	0.100	0.155	0.339		
ENO	0.850	1.220	0.623		
TUB	0.420	0.772	0.428		
**15**°**C infected + uninfected**					
ACT	0.200	0.296			
AK	0.300	**0.095**	**0.134**		
CO1	0.300	0.246			
EF1	0.160	**0.257**	**0.134**	**0.046**	**0.161**
ENO	1.060	1.064		**0.046**	**0.161**
TUB	0.620	0.924			
**35**°**C uninfected**					
ACT	0.860	0.775	0.448	**0.103**	**0.036**
AK	0.610	**0.080**	**0.797**	**0.103**	**0.036**
CO1	0.200	1.000	0.539		
EF1	0.550	0.080	0.797		
ENO	1.010	1.073	0.570		
TUB	0.130	0.421	0.342		
**35**°**C infected + uninfected**					
ACT	0.770	0.404		**0.442**	**0.165**
AK	0.610	0.369		**0.549**	**0.276**
CO1	0.700	0.443			
EF1	0.380	**0.122**	**0.121**	**0.459**	**0.163**
ENO	1.320	0.937			
TUB	0.760	**0.187**	**0.121**	**0.104**	**0.036**
**25**°C **infected**					
ACT	0.280	**0.056**	**0.080**	**0.695**	**0.253**
AK	0.530	0.223		**0.725**	**0.337**
CO1	0.370	0.310			
EF1	0.310	**0.140**	**0.080**	**0.671**	**0.314**
ENO	0.520	0.360			
TUB	0.490	0.238		**0.664**	**0.282**
**25**°**C infected + uninfected**					
ACT	0.520	0.347		**0.309**	**0.106**
AK	0.520	**0.365**	**0.184**	**0.309**	**0.106**
CO1	0.380	0.409			
EF1	0.360	**0.294**	**0.184**		
ENO	0.630	0.554			
TUB	0.590	0.338			
**15**°**C, 25**°**C, 35**°**C infected + uninfected**					
ACT	0.510	0.420		**0.413**	**0.144**
AK	0.490	**0.377**	**0.309**	**0.553**	**0.270**
CO1	0.480	0.657			
EF1	0.330	**0.439**	**0.309**	**0.401**	**0.144**
ENO	0.900	0.825			
TUB	0.650	0.591			

At 25°C, uninfected midgut samples are as described in Table 4.

The geNorm analysis found that the AK and CO1 were the best combination of genes for normalizing the expression of the sample from *T*. *leucotreta* exposed to low temperature stress (15°C) for 48 hours (Table 5). Normfinder selected AK as the overall best-performing gene followed by ACT, EF1, CO1 and TUB. Normfinder rejected ENO as a possible reference gene due to its stability value above 1. Bestkeeper rejected ENO as a possible reference gene due to a SD above 1. When the *T*. *leucotreta* virus-infected samples kept at low temperature were analysed with low temperature controls. geNorm selected ENO and EF1 as the best gene combination for normalization. Normfinder also selected EF1, although combined with AK. ENO was rejected by Normfinder as a potential reference gene. Bestkeeper also rejected ENO and TUB but found all other genes acceptable.

With *T*. *leucotreta* exposed to high temperature (35°C) stress, only two genes, ACT and AK, were required by geNorm for accurate normalization (Table 5). AK was also selected by Normfinder as the best performing gene. Normfinder ranked the genes from most stable to least stable as AK, EF1, TUB, and ACT with CO1 and ENO being excluded as possible reference genes. ENO was also rejected by Bestkeeper as it showed a SD above 1. Combining the high temperature virus-infected samples with the high temperature control samples, reduced overall gene stabilities. geNorm required ACT, AK, EF1, and TUB in conjunction to satisfy normalization of the samples. Normfinder paired EF1 and TUB as the best gene combination and rejected ENO as a potential reference gene. Bestkeeper rejected ENO and TUB as potential reference genes but all other genes satisfied the requirements of this software (Tables 2–5). geNorm analysis of *T*. *leucotreta* virus-infected samples at 25°C for 24, 48 and 72 hours post-infection selected ACT, AK, EF1 and TUB genes for normalization. Although the genes selected had individual M values above the 0.5 threshold, when combined they provided suitable stability. ACT and EF1 were selected as the best paired genes by Normfinder with the remaining genes still satisfying reference gene stability criteria although all genes had low SD. Combining the *T*. *leucotreta* virus-infected and uninfected samples at 25°C for 24, 48 and 72 hours post-infection improved the overall gene stability. AK and ACT were selected by geNorm as the best gene combination whereas Normfinder selected AK and EF1.

## Discussion

To our knowledge this is the first study evaluating potential reference genes for insects within the same tribe as well as an insect under biopesticide stress at different temperatures. This is also the first study comparing reference gene selection for species of Tortricidae and their different tissue types.

Although there have been dramatic improvements in the availability of insect genetic information, it is still limited for many non-model species, and sequences are often not available for important genes for these species [10, 39]. It would be advantageous if the same primers could be used to evaluate related species. To determine whether a single primer pair could be used for multiple species in qPCR analysis, we used multiple species for reference gene amplification. The six primer sets for ACT, AK, CO1, EF1, ENO and TUB genes were found to be suitable for *Cy*. *pomonella* and *T*. *leucotreta* while the ACT, AK, EF1 and TUB genes were suitable for *C*. *peltastica*. These primers will also have a high likelihood of being suitable for other closely-related species for further comparative studies where sequence information is not available.

Ideal reference genes are known to differ among species. Previous studies have compared reference gene selection for two species of *Bombus* [9] and lepidopteran species from different families [7]. As expected, more reference genes were found in common for species within the same genus than those from different families. We investigated whether the same reference genes could be used for species from the same tribe. No reference genes were stable enough to be used for all three species together or in pairs. CO1 expression levels differed dramatically between *Cy*. *pomonella* and *T*. *leucotreta* under the same conditions, with Cq differences of up to 21 Cq. These results are similar to those found previously, which showed that GAPDH differed between lepidopteran families by up to 14 Cq [7]. Therefore, if taxonomic trends for reference gene selection exist for Tortricidae it will be at a lower taxonomic level than tribe. This pattern of sample differences in reference gene selection was also found in the tissue comparisons.

Different tissue types were shown to have a major influence on gene stability. This was seen when the tissue samples from all three species were combined in the analysis. This poor gene stability using multiple tissue types is consistent with previous studies [5, 7–9, 19, 40, 41]. Thus, we assessed reference genes for different tissues separately [5, 22]. None of the genes investigated in this study were suitable for *C*. *peltastica* cuticle tissue normalization and thus alternative genes should be evaluated for that purpose. However, *C*. *peltastica* whole body and midgut tissue samples at 25°C could be normalized using AK and TUB as there was good consensus amongst the three analysis programs. Both midgut and whole body tissue types included for *Cy*. *pomonella* incorporated EF1 as a reference gene, which has previously been found to be stable in different tissue types [9]. AK and EF1 are recommended reference genes for *Cy*. *pomonella* whole body extractions and AK, EF1, ENO and TUB are recommended for *Cy*. *pomonella* midgut tissue. There was a little consensus among the analysis programs for an additional, suitable gene for cuticle analysis of *Cy*. *pomonella*. Therefore, it is suggested alternative genes not included in this study are evaluated for potential combination with EF1 for normalization of *Cy*. *pomonella* cuticle tissue. For *T*. *leucotreta* whole body tissue required ACT and CO1 and cuticle tissue required AK and EF1 for normalization. Midgut tissue analysis for *T*. *leucotreta* found TUB to be a stable gene but as accurate normalization requires genes to be both stable and well paired, the best gene combination was found by geNorm to be AK and EF1.

Major differences were seen in the same tissue type under different stress conditions in *T*. *leucotreta* confirming that tissue type is not the only factor to consider in reference gene selection (Tables 2–5). When *T*. *leucotreta* midgut tissue samples of uninfected larvae were exposed to 35°C for 48 hours, the analysis showed that only two genes were required for normalization. However, when virus-infected samples at 35°C were also included in the analysis, four genes were required for normalization. This is further evidence to highlight the importance of testing reference genes under the specific conditions of the experiment. Stability could be affected in unexpected ways. At 15°C AK was shown to be the most stable gene, paired with CO1. This result confirms a previous finding [5] that AK performs well under low temperatures. Although EF1 has been found to be a stable gene in insects at high temperatures [8, 40, 42], our results showed that ACT and AK were the best combination of genes even though EF1 was still suitable for normalization. However, when virus-infected samples were assessed at high temperature EF1 was required for normalization, in combination with ACT, AK and TUB. When all temperature and virus-stress conditions were combined ACT, AK and EF1 was found to provide the best normalization for *T*. *leucotreta* midgut tissue. Only two studies on reference gene evaluation have been performed using insect viruses. These studies found stable expression of ACT [1] and EF1[41] during the virus infection. In the light of these results, and those produced in this study, it is recommended that ACT, AK and EF1 are used for normalization of virus-infected *T*. *leucotreta* larvae at different temperatures.

In summary, this study successfully identified reference genes for a number of different tissue types for three species of Tortricidae, which provides a foundation for future Tortricidae gene expression research. Although controversial results were sometimes obtained with different algorithms, different tissue types usually required a different suit of reference genes for each species. For some conditions evaluated, more genes are needed to correctly normalise expression. We showed that the same primers can be used to successfully amplify gene regions from different species when assessing reference stability, although universal primers for amplification in the Tortricidae were only found for four of the six genes analysed. We identified stable reference genes for use in *T*. *leucotreta* gene expression studies focusing on the change in expression of CrleGV-SA-infected midgut tissue at different temperatures and times.

## Supporting Information

S1 Table Species and sequences used to design primer pairs for six genes in three species of Tortricidae. (XLSX)

S2 Table Nucleic acid concentrations and purity values for samples analysed using qPCR. (XLSX)
